# A Glance at the Practice of Pediatric Teledermatology Pre- and Post–COVID-19: Narrative Review

**DOI:** 10.2196/34228

**Published:** 2022-05-17

**Authors:** Valencia Long, Nisha Suyien Chandran

**Affiliations:** 1 Division of Dermatology, Department of Medicine, National University Hospital National University Health System Singapore Singapore; 2 Yong Loo Lin School of Medicine National University Singapore Singapore Singapore

**Keywords:** teledermatology, telehealth, telemedicine, pediatric teledermatology, COVID-19, pandemic, dermatology, pediatric, children

## Abstract

**Background:**

The COVID-19 pandemic has accelerated the use of pediatric teledermatology, with centers showing increased uptake of teledermatology. Pediatric patients possess unique characteristics that pose different challenges with teledermatology compared to adults, in turn affecting the feasibility and uptake of pediatric teledermatology in the community.

**Objective:**

This narrative review summarizes the evolution of pediatric teledermatology from pre–COVID-19 pandemic times to the post–COVID-19 period.

**Methods:**

A search of MEDLINE, PubMed, and Embase was performed for original articles written in English and published from December 1, 2019, to April 1, 2022.

**Results:**

A total of 22 publications were included.

**Conclusions:**

Teledermatology will continue to play an increasing role in the management of skin diseases. A mindset shift in the types of conditions deemed suitable for pediatric teledermatology is needed.

## Introduction

The COVID-19 pandemic has accelerated the use of pediatric teledermatology, with centers showing increased uptake of teledermatology. Pediatric patients possess unique characteristics that pose different challenges with teledermatology compared to adults, in turn affecting the feasibility and uptake of pediatric teledermatology in the community. As compared to more self-directed adult patient care, caregivers tend to be more deeply involved in the care of the pediatric patient, with Naka et al [1] having suggested that adopting a family-oriented approach to care and communication is essential. Physicians are obliged to conduct a safe, child-friendly, family-centric consult across a virtual platform when teledermatology for children is used. The limitations and difficulties intrinsic to pediatric teleconsultation may be compounded by obstacles in obtaining details of the full pediatric medical history (including details on prenatal, birth, and developmental history), which can be time-consuming [1]. Heavy reliance on caregivers as sources of information in relation to the child may inadvertently lead to inaccuracy and bias. Pediatric patients, compared to their adult counterparts, are also protected by additional laws governing health care delivery—hence, ethical and legislative-related concerns may escalate when dealing with a pediatric patient [1].

The COVID-19 pandemic has given new impetus for pediatric teledermatology to be embraced as infection control, physical distancing measures, and reduction in hospital attendances post–COVID-19 necessitate more remote yet viable options for pediatric care. This narrative review summarizes the evolution of pediatric teledermatology from pre–COVID-19 pandemic times to the post–COVID-19 period.

## Methods

A search in MEDLINE, PubMed, and Embase was performed for original articles written in English and published from December 1, 2019, to April 1, 2022. Articles were evaluated by reviewing their titles and abstracts for relevance. Articles that lacked relevance to pediatric teledermatology either pre- or post–COVID-19 were excluded. Articles that reported on the practice, outcomes, and experiences with pediatric teledermatology both pre- and post–COVID-19 were used in this narrative review. The search terms used were “paediatric teledermatology,” “telemedicine,” “telehealth,” “virtual,” “videoconferencing,” and “teleconferencing.”

## Results

A total of 22 publications were included.

## Discussion

### Modes of Pediatric Teledermatology Pre- and Post–COVID-19

Pediatric teledermatology can be delivered via synchronous, asynchronous, and hybrid means [2]. Synchronous teledermatology involves real-time/live video teleconferencing, while asynchronous teledermatology, including direct-to-consumer applications, involves store-and-forward platforms transmitting submitted images from the patient, caregiver, or other nondermatological physicians. Hybrid platforms were a mix of the aforementioned modes [2].

A 2015 survey of US dermatologists reported up to 89% of pediatric dermatologists having experience with teledermatology, using store-and-forward platforms, synchronous live consults, and hybrid platforms [2]. Direct-to-consumer pediatric virtual urgent care can be beneficial for viewing the child in the setting of their own home, enabling more reliable observation and assessment of the child’s behaviors and condition. A post–COVID-19 study in a large academic medical center reported that pediatric emergency virtual urgent care could be conducted via telephone calls, allowing practitioners to manage pediatric concerns that were COVID-19–related (36%), dermatologic (15%), and trauma-related (10%) [3]. The authors cited a 4-fold increase in pediatric emergency virtual urgent care volume post–COVID-19 compared to the same period pre–COVID-19, underscoring the need for pediatric telehealth during the pandemic.

There is scope for creativity and optimization of teledermatology delivery modes post–COVID-19 to improve patient access and uptake. Virtual reality (VR) technology, where a headset can be worn by the teleprovider in various settings and even on-the-go, removes logistical barriers for teleproviders. Adoption of VR technology for parents/families may, on the other hand, be challenging. It may be costly to ensure adequate access with provision of VR technology to families. Parents may also face physical difficulties with using VR for the roving active child. We propose that VR consultation would be better suited for the older pediatric patient who may be able to sit still for the duration of the consultation. Families/parents offered VR consultation should ideally be financially stable or adequately supported.

The challenges physicians face with teledermatology consults pre- and post–COVID-19 are likely to remain similar, with the potential inability of physicians to make the correct dermatological diagnosis as a result of information inadequacy (insufficient history obtained over virtual consult, limited ability to examine the patient thoroughly, poor quality clinical images, limited ability to use bedside equipment such as dermoscopy to examine lesions).

We suggest that when there are difficulties, physicians can consider using hybrid platforms to improve the accuracy of teledermatology. For example, a caregiver could photograph a young child’s skin lesions while the child was asleep to enable better quality photographs without the child’s movement. A synchronous teledermatology consultation could then improve evaluation of the skin lesions. This use of hybrid platforms is similarly useful if there are challenges in the child being present for the entire length of the consult.

Privacy concerns can be ameliorated using photographs curated by the caregiver in lieu of real-time/live visual examination of sensitive body sites [4]. For older children who may have private information to divulge directly to health care practitioners, their parents/caregivers could be invited to leave the teledermatology consult room. This practice would mirror traditional face-to-face consultation.

The importance of high-quality photographs of skin disorders cannot be understated, with various studies highlighting the use of clinical photography in teledermatology [1,2,5,6]. With advancements in digital photography and storage/transmission technology, obtaining photographs assists not merely clinical care via teledermatology but also education, research, and patient documentation. With store-and-forward platforms, good photography forms the basis of the consult—where clinical photos inform on the evolution of a skin disease and assist in more objective assessments of a patient when different doctors are involved in patient care such as in larger public hospitals [5]. A scoping review by Kim and Sivesind [7] had also demonstrated that patients adopt positive attitudes toward medical photography. Most patients had expressed that medical care could be improved in the clinical setting with photography [7].

In the transmission of clinical images, data protection is paramount. The personal data of patients could be protected by pseudo anonymization (limiting access to authorized personnel), entity authentication, and data encryption [8].

### Conditions Managed by Teledermatology Pre- and Post–COVID-19

Pre–COVID-19, diagnoses during pediatric teledermatology consults were similar to in-person visits, including inflammatory dermatoses such as atopic dermatitis; pityriasis alba; acne; xerosis; infective issues such as molluscum contagiosum; verruca vulgaris; benign and malignant tumors including melanocytic nevi, infantile hemangiomas (IH), and other skin tumors; wound care; pigmentary conditions (including tinea versicolor, vitiligo, postinflammatory hyperpigmentation, and hypopigmentation); and alopecia [2,9,10]. Prior to the COVID-19 pandemic, a cross-sectional Brazilian study [11] had commented that 63% of lesions in the primary care settings could be managed via store-and-forward teledermatology without the need for an in-person visit, as only 1% of cases required biopsy.

Post–COVID-19, reported pediatric teledermatology consult diagnoses included atopic dermatitis, Gianotti Crosti Syndrome, intertrigo, photodermatitis, acne, adnexal disorders, alopecia, IH, skin infections/infestations, molluscum contagiosum, verruca vulgaris, melanocytic nevi, pigmentary disorders, and psoriasis [2,11].

Apart from the consensus statement released by the Society of Pediatric Dermatology on the management of IH, suggesting that follow-up visits for IH could be safely performed via synchronous or asynchronous means, there is relative paucity of teledermatology practice guidelines for the other pediatric conditions. Prior to the COVID-19 pandemic, strides in teledermatology had lagged at least in part from a lack of confidence in telediagnoses and fears of missing or delaying critical diagnoses [12]. With the ongoing pandemic, and in line with the American Academy of Pediatrics, clinical practice guidelines could give practitioners a collective personal experience on teledermatology management. This may aid in assauging diagnostic apprehension.

Havele et al [13] retrospectively reviewed 1110 patient-provider live video consults and 89 store-and-forward, provider-to-provider pediatric teledermatology consults during the COVID-19 pandemic and described that dermatologists faced different issues in terms of connectivity, video quality, and photograph quality when managing myriad conditions. In this study, the management of alopecia was associated with issues with video and photograph quality. Pre–COVID-19, a 2017 randomized controlled trial of 40 patient-parent dyads at a US pediatric dermatology clinic found high concordance between photograph-based and in-person diagnosis but had also highlighted that alopecia and neoplasms tended to have lower concordance rates between photographs and in-person diagnoses [14]. We suggest that providers could consider using hybrid platforms integrating both synchronous and asynchronous communication. Close-up photos with applied dermoscopy may assist.

As the pandemic lingers on, pediatric teledermatology should evolve to cater to a wider range of conditions. Contact dermatitis and mask-induced acne arising from pandemic-related health precautions could be effectively managed via teledermatology [15]. Pediatric teledermatology should be expanded to slowly include the review of genodermatoses, which had already in pre–COVID-19 times seen a scarcity of expert pediatric dermatologists, with multidisciplinary clinics being typically restricted to large cities [2].

### Reimbursement and Medicolegal Implications With Teledermatology Pre- and Post–COVID-19

One of the most substantial barriers to widespread adoption of teledermatology is likely related to the lack of reliable systems for reimbursement. A large survey carried by the American Academy of Dermatology (AAD) in 2021 of 5000 participants revealed that reimbursement concerns formed the majority (69.8%) of all reported barriers [16]. Teledermatology services globally operate via a variety of business models (which need to be sustainable) including per-case service contracts, direct-to-consumer, and standard fee-for-service reimbursement [17].

Prior to the COVID-19 pandemic in the United States, private insurers offered reimbursement for teledermatology as an alternative to self-pay and federal health insurance programs such as Medicare and Medicaid. However, such policies varied between states and payer status. This was similarly poorly delineated in the realm of teledermatology in many centers worldwide, thus disincentivizing uptake. Since the COVID-19 pandemic, the AAD has advised that Medicare could allow reimbursement of telehealth services rendered via telephone with payment for telephone-only encounters being reimbursed at the same rate as in-person (new/established) office visits. Rates are based on the national Medicare Physician Fee Schedule [18].

Although the pandemic has likely improved flexibility of reimbursement for telephonic consults, reimbursement policies for store-and-forward services would similarly likely benefit from review. The comparatively low reimbursement rates for store-and-forward teledermatology consults may undervalue the time and expertise of the practicing dermatologists. Practitioners may instead choose to use store-and-forward platforms for image control and quality while completing consults using videoconferencing. Ensuring reasonable reimbursement for multimodal/combination methods of teledermatology consults can improve both uptake among dermatologist and patient-centric outcomes.

Medicolegal implications associated with teledermatology may pose significant challenges to uptake, with the AAD survey highlighting 27% of participants having concerns with malpractice/liability [16]. To date, the extent of legal responsibility in cases of incorrect/delayed diagnosis remains ambiguous, and malpractice risk is not generally well characterized. Although a 2019 study [19] cited no reported cases of medical malpractice against direct-to-patient telemedicine, the fact that patients’ privacy could be compromised at various checkpoints of image capture, transmission, and storage remains concerning. In the United States, the Health Insurance Portability and Accountability Act (HIPAA) governs the compliance of clinical images to follow appropriate security precautions, with providers who fail to do so being subjected to legal penalties. Although temporarily waived as part of the initial response to the COVID-19–imposed public health emergency [20], the unlikely continuation of this waiver in postpandemic times reminds dermatologists that good clinical practice entails documented patient consent for all clinical images, providing explanations on the use of images, and ensuring HIPAA-compliant security in storage and transmission.

### Cultural and Socioeconomic Considerations for the Implementation of Pediatric Teledermatology Post–COVID-19

A US study comparing pediatric teledermatology visits scheduled post–COVID-19 with in-person appointments in the same period pre–COVID-19 reported that certain demographic groups such as Spanish-speaking patients were less likely to have teledermatology visits [21]. Another single center US study [22] demonstrated via multivariate analyses that independent factors associated with lower rates of telemedicine use were patients identifying as Black/African American and having a non-English preferred language. In this study, patients on public insurance were also found to have significantly lower odds of telemedicine use despite widely expanded telehealth coverage by US health insurance plans. Low-income households may experience gaps in access to technology and internet connectivity that are requisites for teledermatology visits. Differential digital literacy and connectedness among cultural and socioeconomic groups can create inequity in pediatric teledermatology uptake and care delivery. Physicians need to be sensitive and remain current about patient communication preferences. For instance, for patients who may be less likely to engage in patient portal communications, other modes of communication via SMS text messaging or video calls could be alternative modes of telehealth delivery for underserved populations.

There are also disparities in trust toward dermatologists providing teledermatology among patients of different racial groups. Kim and Sivesind [7], in a scoping review, revealed that patients of Latin and African American descent had expressed less trust in the utility of medical photography to improve patient care, compared to patients who were Asian and White. Ethnic disparities in patient perceptions may need to be taken into consideration by practitioners to improve the teledermatology experience for socially marginalized patients. This could be done by using nonphysician photographers and clinic-owned cameras, and by improving patient education surrounding the safety of electronic medical record phone apps [7].

### Conclusion

Teledermatology will continue to play an increasing role in the management of skin diseases. A mindset shift in the types of conditions deemed suitable for pediatric teledermatology is needed. Practitioners should also be aware of the various modes of teledermatologic delivery to select the most appropriate mode that is sensible for both practitioner and patient, taking into account potential socioeconomic challenges and cultural preferences. Given that previous studies have shown that both store-and-forward and live interactive teledermatology could be diagnostically comparable, hybrid models may further help ameliorate physician-patient diagnostic and logistical difficulties. Further study into the comparable diagnostic accuracy of teledermatology in skin conditions in pediatric versus adult patients will help highlight advantages and shortfalls of teledermatology in different age groups. A similar comparison between acute emergent versus chronic dermatoses in the pediatric age group will be useful. A concerted effort to characterize the practice of teledermatology in the post–COVID-19 era will allow practitioners to fine-tune and get comfortable with this modality.

## Abbreviations

AAD: American Academy of Dermatology
HIPAA: Health Insurance Portability and Accountability Act
IH: infantile hemangiomas
VR: virtual reality
